# Promoting the use of the PI-QUAL score for prostate MRI quality: results from the ESOR Nicholas Gourtsoyiannis teaching fellowship

**DOI:** 10.1007/s00330-022-08947-5

**Published:** 2022-06-30

**Authors:** Francesco Giganti, Alexander P. Cole, Fiona M. Fennessy, Timothy Clinton, Pedro Lopes Da Frota Moreira, Mariana Costa Bernardes, Carl-Fredrik Westin, Deepa Krishnaswamy, Andriy Fedorov, Daniel A. Wollin, Bjoern Langbein, Nicola Frego, Muhieddine Labban, Joy S. Badaoui, Steven L. Chang, Logan G. Briggs, Junichi Tokuda, Alessandro Ambrosi, Alex Kirkham, Mark Emberton, Veeru Kasivisvanathan, Caroline M. Moore, Clare Allen, Clare M. Tempany

**Affiliations:** 1 Department of Radiology, University College London Hospital NHS Foundation Trust, London, UK; 2 Division of Surgery & Interventional Science, University College London, 3rd Floor, Charles Bell House, 43-45 Foley St., W1W 7TS, London, UK; 3 Division of Urological Surgery, Centre for Surgery and Public Health, Brigham and Women’s Hospital, Harvard Medical School, Boston, MA USA; 4 Department of Radiology, Brigham and Women’s Hospital, Harvard Medical School, Boston, MA USA; 5 School of Medicine, Vita-Salute San Raffaele University, Milan, Italy; 6 Department of Urology, University College London Hospital NHS Foundation Trust, London, UK

**Keywords:** Urogenital neoplasms, Prostatic neoplasms, Magnetic resonance imaging, Biopsy

## Abstract

**Objectives:**

The Prostate Imaging Quality (PI-QUAL) score is a new metric to evaluate the diagnostic quality of multiparametric magnetic resonance imaging (MRI) of the prostate. This study assesses the impact of an intervention, namely a prostate MRI quality training lecture, on the participant’s ability to apply PI-QUAL.

**Methods:**

Sixteen participants (radiologists, urologists, physicists, and computer scientists) of varying experience in reviewing diagnostic prostate MRI all assessed the image quality of ten examinations from different vendors and machines. Then, they attended a dedicated lecture followed by a hands-on workshop on MRI quality assessment using the PI-QUAL score. Five scans assessed by the participants were evaluated in the workshop using the PI-QUAL score for teaching purposes. After the course, the same participants evaluated the image quality of a new set of ten scans applying the PI-QUAL score. Results were assessed using receiver operating characteristic analysis. The reference standard was the PI-QUAL score assessed by one of the developers of PI-QUAL.

**Results:**

There was a significant improvement in average area under the curve for the evaluation of image quality from baseline (0.59 [95 % confidence intervals: 0.50–0.66]) to post-teaching (0.96 [0.92–0.98]), an improvement of 0.37 [0.21–0.41] (*p* < 0.001).

**Conclusions:**

A teaching course (dedicated lecture + hands-on workshop) on PI-QUAL significantly improved the application of this scoring system to assess the quality of prostate MRI examinations.

**Key Points:**

• *A significant improvement in the application of PI-QUAL for the assessment of prostate MR image quality was observed after an educational intervention.*

• *Appropriate training on image quality can be delivered to those involved in the acquisition and interpretation of prostate MRI.*

• *Further investigation will be needed to understand the impact on improving the acquisition of high-quality diagnostic prostate MR examinations.*

**Supplementary Information:**

The online version contains supplementary material available at 10.1007/s00330-022-08947-5.

## Introduction

Prostate magnetic resonance imaging (MRI) examinations must be of high quality to allow for accurate interpretations. Low diagnostic quality examinations will increase uncertainty in MRI decision-making [1]. Against this backdrop, the Prostate Imaging Reporting and Data System (PI-RADS) standards Versions 2.0 and 2.1 were developed to include a set of minimal technical requirements for the acquisition of good-quality MRI of the prostate [2, 3]. Additionally, two panels of experts have advocated the creation of standardised quality criteria for the evaluation of the image quality of prostate MRI [4, 5].

The Prostate Imaging Quality (PI-QUAL) score for prostate MRI represents the first standardised scoring system that evaluates image quality using objective technical criteria together with subjective visual criteria from the images [6].

PI-QUAL score of 1 means that all MR sequences are below the minimum standard of diagnostic quality, a PI-QUAL score of 3 indicates that the study is of sufficient diagnostic quality (as at least two MR sequences taken together are of diagnostic quality), and a PI-QUAL score of 5 implies that all sequences are of optimal diagnostic quality (i.e., all clinically significant lesions can be ruled in AND ruled out) (Table 1).

**Table 1:** Assessment of the diagnostic quality of multiparametric MRI scans using the PI-QUAL score

PI-QUAL score	Criteria	Clinical implications
1	All mpMRI sequences are below the minimum standard for diagnostic quality	It is NOT possible to rule in all significant lesions * It is NOT possible to rule out all significant lesions *
2	Only one mpMRI sequence is of acceptable diagnostic quality	
3	At least two mpMRI sequences taken together are of acceptable diagnostic quality	It is possible to rule in all significant lesions It is NOT possible to rule out all significant lesions
4	Two or more mpMRI sequences are independently of optimal diagnostic quality	It is possible to rule in all significant lesions It is possible to rule out all significant lesions
5	All mpMRI sequences are of optimal diagnostic quality	

*Therefore, reports should not include PI-RADS or Likert scores

Legend. *PI-QUAL* Prostate Imaging QUALity; *mpMRI* multiparametric magnetic resonance imaging; *PI-RADS* Prostate Imaging Reporting and Data System

The importance of reader training and experience in prostate MRI is both intuitive and evident from the literature [7, 8]. As the volume of prostate imaging grows, a larger and larger number of examinations are being performed in centres all over the world. Many sites are beginning for the first time, without a lot of experience in this examination. Dedicated imaging site and personnel efforts are required to ensure high quality.

The first step in reviewing a prostate MR exam is a critical review of its quality. In the current prostate cancer pathways, many patients will be seen by physicians with a prior prostate MR exam already performed elsewhere [9]. So, urologists, oncologists, and others without formal radiology training must be able to ascertain the overall quality of an exam and the resulting diagnostic report. Some men may require a repeat MR examination prior to deciding on treatment. Therefore, the ability to evaluate the quality of prostate MR studies is a crucial skill for clinicians treating prostate cancer.

The Nicholas Gourtsoyiannis Teaching Fellowship, established by the European School of Radiology (ESOR), is aimed at radiologists who wish to enhance their teaching and training skills by delivering lectures and undertaking interactive workshops. For the year 2021, the fellowship has been awarded to two separate projects on prostate MRI.

We report here the results from the second project, which focussed on how an educational intervention can be used to understand and apply the PI-QUAL score to evaluate the quality of prostate MRI images [6].

Our hypothesis was that dedicated training in assessing image quality by using the PI-QUAL score would significantly improve the ability of participants with different levels of experience and training backgrounds in determining the adequacy of the images.

## Materials and methods

The 2021 fellowship recipient (F.G.) is a Consultant Radiologist with a particular interest in genitourinary imaging and highly experienced in prostate MRI (i.e., reporting more than 2,500 prostate MR scans per year), and who is also one of the developers of the PI-QUAL score [6].

**Fig. 1:**
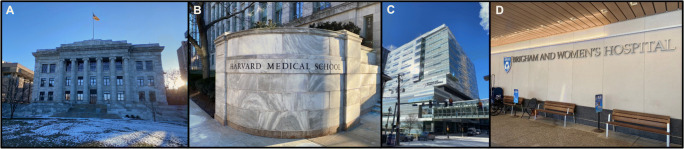
Harvard Medical School (**A**, **B**) and main entrance of Brigham and Women’s Hospital (**C**, **D**) in Boston, Massachusetts, USA. The images were taken during the teaching fellowship in January 2022

The second teaching fellowship took place at Harvard Medical School/ Brigham and Women’s Hospital in Boston, MA, USA (Fig. 1), between January 10 and January 16, 2022.

### Setting and participants

Study participants with different levels of experience in prostate MRI and training backgrounds (but all linked to the MR pathway for the diagnosis of prostate cancer) were invited from the departments of radiology and surgery/urology. The only prerequisites for participation were to have general knowledge of the anatomy of the prostate on MRI and to have been exposed to multiparametric MR images of the prostate before the course.

All participants were unaware of the specifics of the PI-QUAL assessment procedure and how to apply it.

### MR examinations

All MR examinations included T2-weighted, diffusion-weighted, and dynamic contrast-enhanced sequences and it is important to mention that they were not always fully compliant with the PI-RADS recommendations (i.e., technical details), reflecting the heterogeneity of the conduct of prostate MRI across different centres [9]. The scans had been performed between November 2018 and September 2021 without an endorectal coil. All MR exams and images had been previously anonymised and uploaded onto a dedicated DICOM viewer. All participants reviewed all sets of cases and were blinded to the magnet vendor, field strength, and all clinical data.

Due to the institutional restrictions in place at the time of the fellowship (following the rapid spread of the Omicron variant of COVID-19), the teaching course was conducted on-site in Boston, USA, but it was decided to hold the lecture and the workshop also via a video-meeting platform to allow the attendees to join the lecture and the workshop remotely.

### Specific of the educational intervention:

**Fig. 2:**
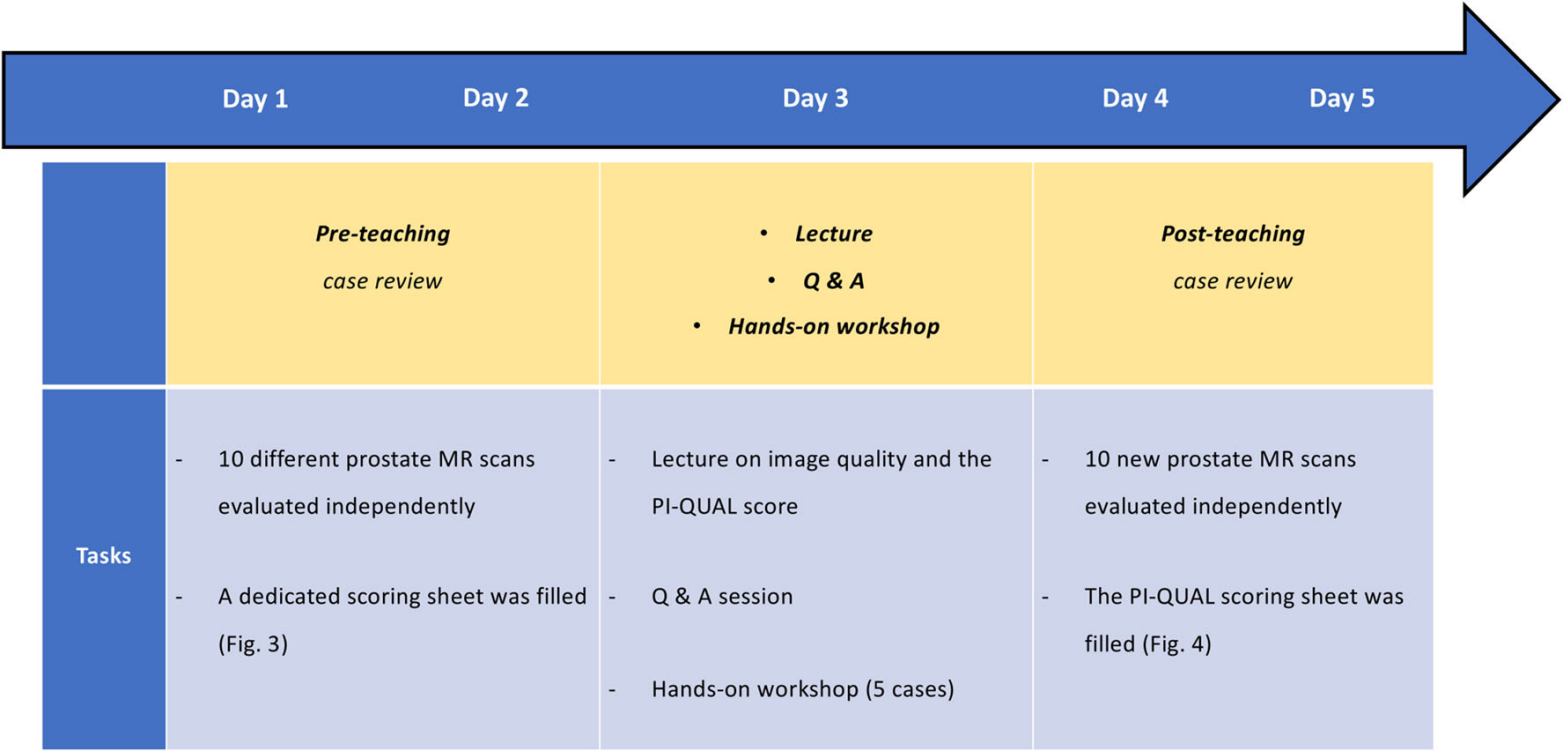
Chronologic framework of the teaching fellowship

Figure 2 shows the framework of each step of the teaching fellowship during the week.

- *Day 1 and day 2 (pre-teaching case review)*

**Fig. 3:**
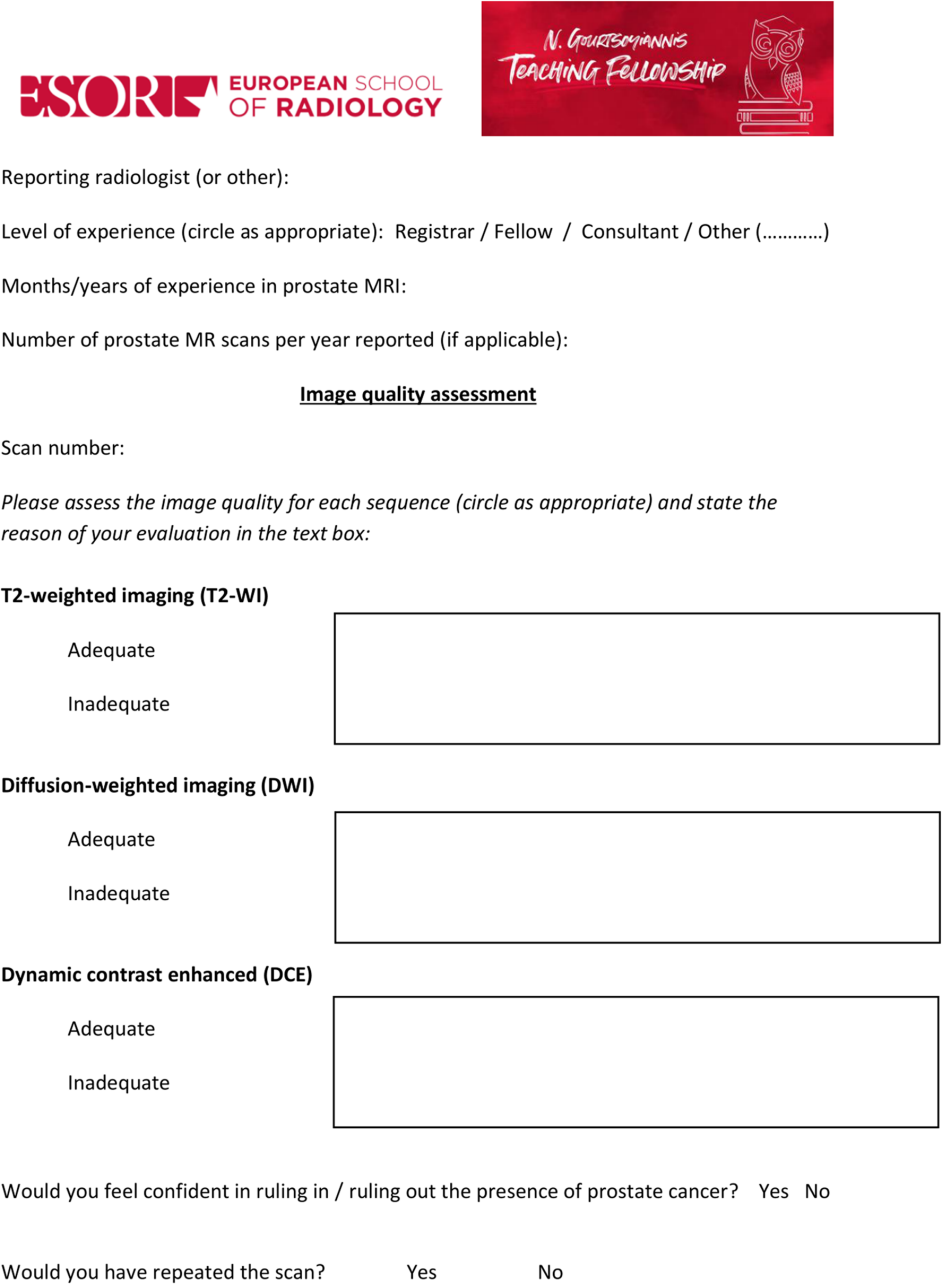
Scoring sheet for pre-teaching scans

In the two days prior to the lecture, the participants were asked to go through a set of different MR scans of the prostate independently. They filled a scoring sheet (Fig. 3) that was specifically created for this project by the course director (F.G.) in which they evaluated the quality of each of the three sequences (i.e., T2-weighted imaging, diffusion-weighted imaging and dynamic contrast-enhanced sequences). They were also asked to state if they would have repeated the scan due to poor image quality. No further instructions or guidelines were given, and the participants relied only on their previous knowledge in prostate MRI. Although we kept track of time, participants were allowed to work at their own pace to interpret the scans. The scoring sheets were returned and collated before the lecture and the workshop.

- *Day 3 (lecture + hands-on workshop)*

All participants attended the lecture (1 h), which was followed by a Q and A session (30 min) in which they were encouraged to ask questions to improve their understanding of the subject.

#### Lecture framework

The lecture, whose title was: “*Prostate MR and image quality: it is time to improve*,” consisted of two modules:

- Module 1-*Prostate MRI: introduction to (i) the different MR sequences, (ii) the anatomy of the prostate gland, and (iii) the appearance of prostate cancer on MRI*
- Module 2-*Review of the currently available papers addressing the issue of prostate MRI quality and explanation of the PI-QUAL score using a recently published primer* [10]

#### Hands-on workshop framework

**Fig. 4:**
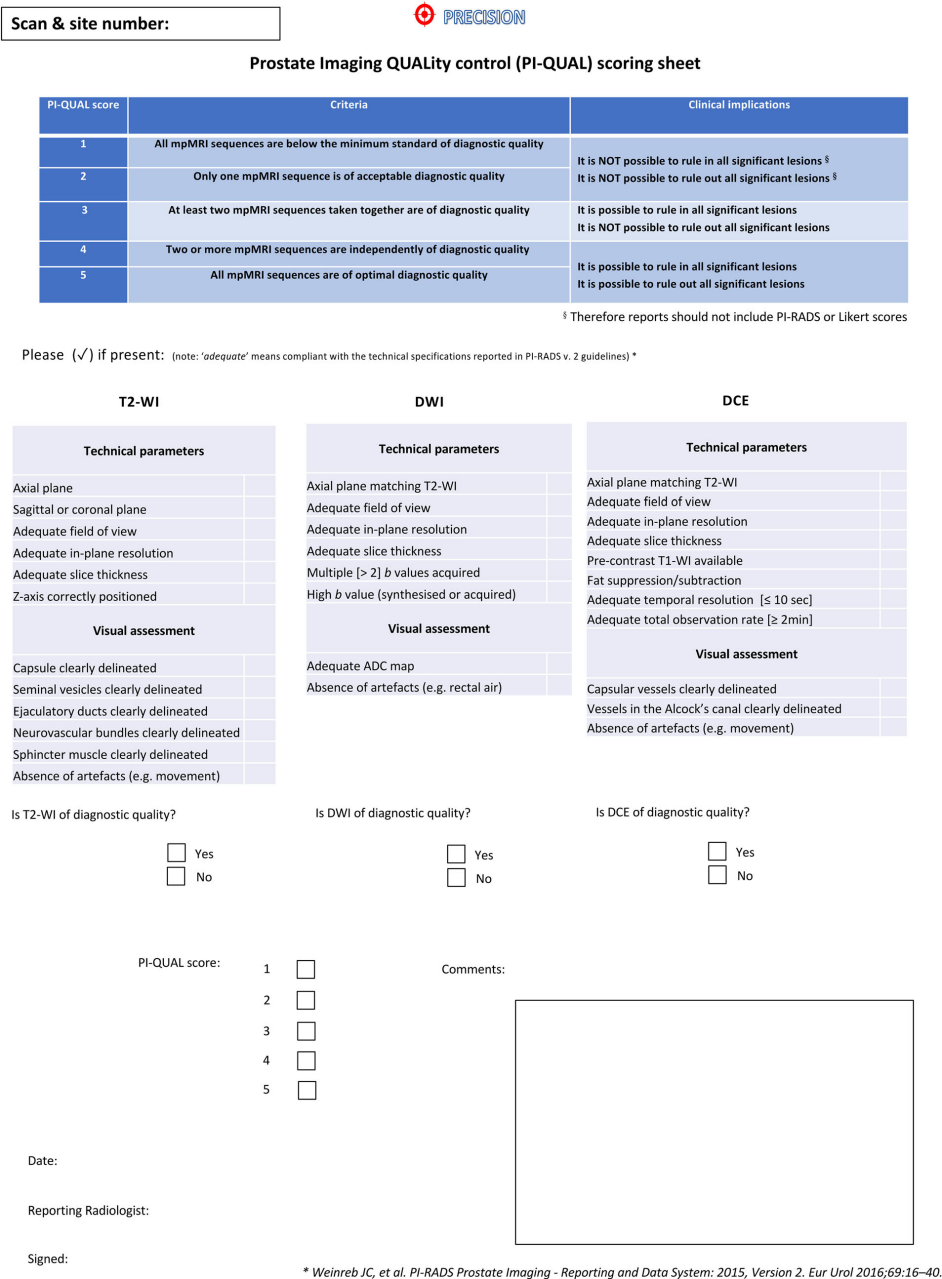
PI-QUAL scoring sheet for post-teaching scans. Reprinted with permission from Elsevier from Giganti F, Allen C, Emberton M, Moore CM, Kasivisvanathan V, for the PRECISION study group. Prostate Imaging Quality (PI-QUAL): A New Quality Control Scoring System for Multiparametric Magnetic Resonance Imaging of the Prostate from the PRECISION trial. Eur Urol Oncol (2020); 3(5):615-619.

The workshop, whose title was: “*The PI-QUAL score: from theory to practice*,” was carried out after the lecture and included hands-on training to familiarise the participants with the PI-QUAL scoring system. During the workshop, which lasted for approximately 1 h, five scans from the pre-training cohort were reviewed and discussed collegially. The participants were taught how to evaluate image quality and assess the PI-QUAL score using the dedicated scoring sheet (Fig. 4) and the PI-QUAL primer. [10]

- *Day 4 and day 5 (post-teaching case review)*

All participants were asked to evaluate independently the image quality of a new set of 10 cases using the PI-QUAL scoring sheet. At the end of the workshop, their results were collated for analysis.

### Clinical cohort and reference standard

All patients included in this study gave written informed consent to have their images used for research and teaching purposes. No Institutional Review Board approval was needed for this study, as the scans were from different MR systems and MR vendors at different centres and were randomly selected by a research assistant not involved in the study and with no clinical background.

The quality assessment given by the course director was used as the reference standard for the evaluation of the participants’ performance before and after the course.

### Statistical analysis

The primary outcome was the change in the average area under the curve (AUC) for detection of suboptimal and optimal image quality (stratified by PI-QUAL 1-3 *vs* PI-QUAL 4-5) before and after teaching.

The pre-teaching scores given in the dedicated scoring sheet (i.e., inadequate image quality *vs* adequate image quality) were dichotomised into PI-QUAL 1-3 *vs* PI-QUAL 4-5.

Receiver operating characteristic (ROC) curves were based on generalised linear mixed models with participant and case ID taken as random effects. This approach generalises the Obuchowski-Rockette method [11] and is described by Liu et al [12].

For each ROC curve and AUC value, 95% confidence intervals (CIs) were computed by conditional bootstrap resampling (*B* = 50,000 samples).

Exact *p* values were computed by permutation methods to avoid any distributional assumption or asymptotic approximation and considered significant when < 0.05.

All statistical analyses were performed in R v. 4.1.3 (R Foundation for Statistical Computing).

## Results

A total of sixteen participants completed the study. A total of twenty scans from twenty different patients were included. The image analysis sessions consisted of 10 cases in the pre- and 10 cases in the post-intervention groups. Table 2 shows the list of participants with their background and their level of experience in prostate MRI.

**Table 2:** List of participants with their background and their level of experience in prostate MRI interpretation

Participant	Background	Experience in prostate MRI (years)	Number of prostate MR scans seen per year
1	Radiologist, MD	30	1,500
2		20	1,000
3	Computer scientist, PhD	5	100
4		0	< 10
5		1	100
6		5	200
7		0	< 10
8	Urologist, MD	9	200
9		5	300
10		2	100
11		0	< 10
12		0	< 10
13		15	100
14		0	< 10
15		8	100
16	Physicist, PhD	1	100

Legend. *MD* medical doctor; *PhD* doctor of philosophy; *MRI* magnetic resonance imaging; *MR* magnetic resonance

The MR examinations were diverse in vendor and field strength: 8/20 (40%) patients were scanned on Siemens®, 6/20 (30%) on General Electric®, and 6/20 (30%) on Philips® scanners.

Eleven out of twenty studies (55%) were conducted on a 1.5 T and 9/20 (45%) studies were conducted on a 3 T system.

Table 3 shows the MR vendors and systems, magnets and PI-QUAL scores (according to the reference standard) for each scan in the pre- and post-teaching cohorts.

**Table 3:** List of MR vendors and systems, magnets, and PI-QUAL scores (according to the reference standard) for each scan in the pre- and post-teaching cohorts

	MR vendor and system	Magnet (T)	PI-QUAL score
Pre-teaching			
Scan 1	Siemens Skyra	3	4
Scan 2	Siemens Verio	3	3
Scan 3	Siemens Avanto	1.5	4
Scan 4	Philips Intera	1.5	2
Scan 5	Philips Ingenia	1.5	4
Scan 6	GE Signa	1.5	2
Scan 7	Siemens Skyra	3	2
Scan 8	Siemens Skyra	3	2
Scan 9	Siemens Verio	3	4
Scan 10	Siemens Prisma	3	5
Post-teaching			
Scan 1	GE Optima	1.5	4
Scan 2	Philips Ingenia	3	4
Scan 3	Philips Ingenia	3	4
Scan 4	GE Signa	1.5	4
Scan 5	GE Optima	1.5	1
Scan 6	Philips Ingenia	1.5	4
Scan 7	Siemens Sola	1.5	5
Scan 8	GE Optima	1.5	2
Scan 9	GE Optima	1.5	1
Scan 10	Philips Ingenia	3	5

Legend. *MR* magnetic resonance; *T* Tesla; *PI-QUAL* Prostate Image Quality

### PI-QUAL score

In the pre-teaching cohort, 5/10 (50%) scans were scored PI-QUAL 2, one (10%) scan was scored PI-QUAL 3, 3/10 (30%) scans were scored PI-QUAL 4, and for only one (10%) scan all sequences were of optimal diagnostic quality (i.e., PI-QUAL 5), according to the reference standard reported in each PI-QUAL scoring sheet (Supplementary Table 1).

In the post-teaching cohort, 2/10 (20%) scans were scored PI-QUAL 1, one (10%) scan was scored PI-QUAL 2, 5/10 (50%) scans were scored PI-QUAL 4, and 2/10 (20%) scans were scored PI-QUAL 5, according to the reference standard reported in each PI-QUAL scoring sheet (Supplementary Table 2).

### Accuracy in evaluating image quality before and after teaching

**Fig. 5:**
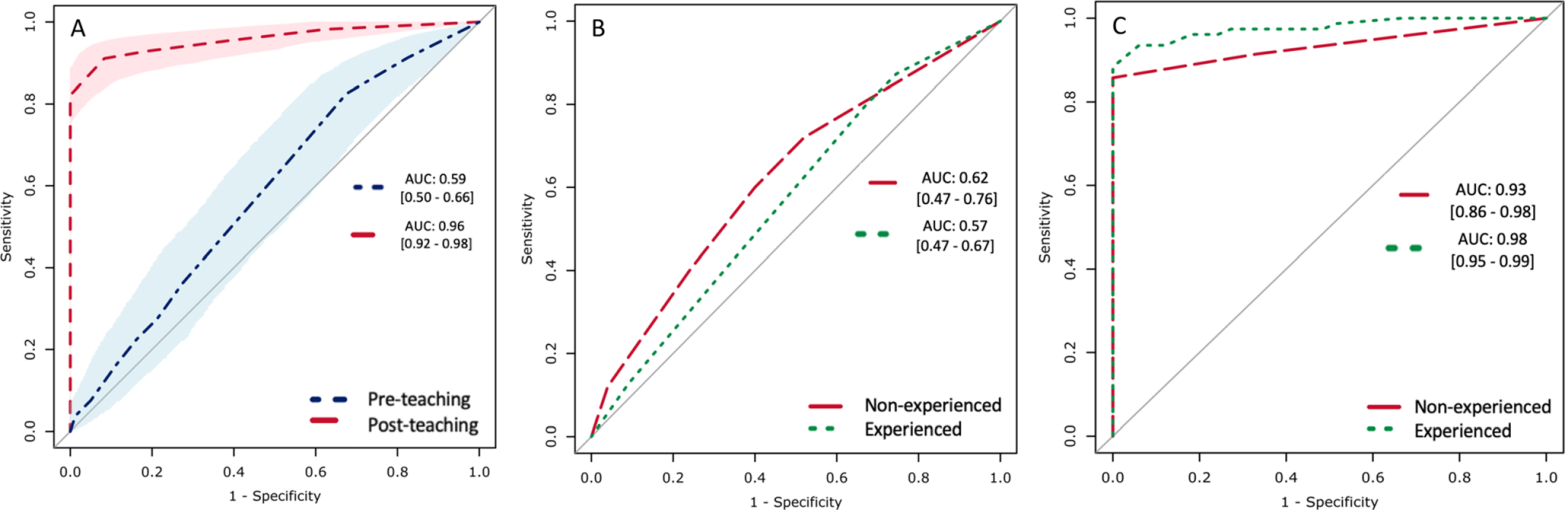
Average area under the curve (AUC) for the evaluation of image quality (suboptimal *vs* optimal) in the pre-teaching (blue, dash-dotted line) and post-teaching (red, dashed line) cohorts, with shaded areas and square brackets representing the 95% confidence intervals (**A**). AUCs for the evaluation of image quality stratified by experience (i.e., < or > than 100 prostate MR scans evaluated) before (**B**) and after (**C**) teaching

There was a significant improvement in the average AUC for the evaluation of image quality (suboptimal *vs* optimal) from pre-teaching (0.59; [0.50-0.66]) to post-teaching (0.96 [0.92-0.98]), an improvement of 0.37 [0.21-0.41] (*p* < 0.001). The ROC curves presented in Fig. 5a summarise the average accuracy levels in evaluating image quality (stratified by PI-QUAL 1-3 *vs* PI-QUAL 4-5) before and after the teaching course.

We also evaluated the AUC in the assessment of image quality stratified by experience before and after teaching. Given the different background of the participants, this was stratified as *non-experienced* participants (i.e., < 100 prostate MR scans seen before the course) and *experienced* participants (i.e., > 100 prostate MR scans seen before the course).

For *non-experienced* participants, the pre-teaching AUC was 0.62 [0.47-0.76] and the post-teaching AUC was 0.93 [0.86-0.98], a difference of 0.31 [0.20-0.38] (*p* < 0.001).

For *experienced* participants, the pre-teaching AUC was 0.57 [0.47-0.67] and the post-teaching AUC was 0.98 [0.95-0.99], a difference of 0.41 [0.30-0.50] (*p* < 0.001).

The ROC curves shown in Fig. 5b (pre-teaching) and 5c (post-teaching) are a visual representation of these findings.

**Fig. 6:**
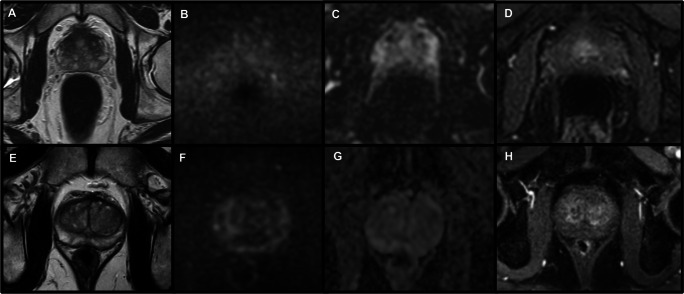
Two cases of prostate MRI from the two cohorts (pre- and post-teaching). The first case [**A** axial T2-weighted imaging; **B** diffusion-weighted imaging (high *b* value: 1,000 s/mm^2^); **C** apparent diffusion coefficient map; **D** dynamic-contrast enhanced sequences] is from the pre-teaching cohort and the reference standard was PI-QUAL 2, as only T2-WI is of sufficient diagnostic quality. Only 7/16 participants gave the correct PI-QUAL score for this scan, with 1/16 giving a PI-QUAL score of 3, 3/16 a PI-QUAL score of 4 and 5/16 a PI-QUAL score of 5. The second case [**E** axial T2-weighted imaging; **F** diffusion-weighted imaging (high *b* value: 2,000 s/mm^2^); **G** apparent diffusion coefficient map; **H** dynamic-contrast enhanced sequences] is from the post-teaching cohort and the reference standard was PI-QUAL 5. All patients scored this scan as of optimal diagnostic quality (i.e., PI-QUAL score 4 or 5; in detail: 9/16 scored PI-QUAL 4 and 7/16 scored PI-QUAL 5)

The PI-QUAL scores given by each participant after the course are presented in Table 4 and two examples of prostate MR examinations (one from the pre-teaching cohort and one from the post-teaching cohort) with their corresponding PI-QUAL scores (according to the reference standard) are presented in Fig. 6.

**Table 4:** PI-QUAL scores given by each participant for each scan after teaching, and reference standard

	Scan 1	Scan 2	Scan 3	Scan 4	Scan 5	Scan 6	Scan 7	Scan 8	Scan 9	Scan 10
PI-QUAL reader 1	**4**	**4**	**4**	**4**	**1**	**4**	**5**	**2**	3	**5**
PI-QUAL reader 2	**4**	**4**	**4**	5	**1**	**4**	2	1	**1**	4
PI-QUAL reader 3	**4**	**4**	**4**	**4**	**1**	**4**	**5**	1	**1**	4
PI-QUAL reader 4	5	**4**	1	**4**	2	**4**	4	1	**1**	4
PI-QUAL reader 5	**4**	**4**	3	**4**	**1**	3	3	1	2	4
PI-QUAL reader 6	**4**	**4**	2	2	**1**	**4**	4	**2**	2	4
PI-QUAL reader 7	5	**4**	1	2	**1**	**4**	4	**2**	**1**	**5**
PI-QUAL reader 8	**4**	**4**	3	3	**1**	**4**	**5**	**2**	**1**	**5**
PI-QUAL reader 9	3	**4**	**4**	3	2	**4**	**5**	1	3	**5**
PI-QUAL reader 10	1	**4**	3	**4**	2	5	**5**	1	2	**5**
PI-QUAL reader 11	**4**	5	2	1	**1**	**4**	**5**	**2**	**1**	4
PI-QUAL reader 12	**4**	**4**	**4**	**4**	**1**	**4**	**5**	1	**1**	4
PI-QUAL reader 13	**4**	**4**	**4**	**4**	2	**4**	4	3	2	4
PI-QUAL reader 14	**4**	**4**	**4**	**4**	**1**	**4**	4	**2**	2	**5**
PI-QUAL reader 15	**4**	**4**	3	**4**	**1**	3	4	1	3	**5**
PI-QUAL reader 16	**4**	**4**	1	**4**	**1**	**4**	**5**	1	2	4
**PI-QUAL reference standard**	**4**	**4**	**4**	**4**	**1**	**4**	**5**	**2**	**1**	**5**

Legend. *PI-QUAL* Prostate Imaging Quality. In boldface are cases concordant with the reference standard

## Discussion

In our study, we have shown that a dedicated teaching course on the application of PI-QUAL to evaluate the quality of prostate MRI is useful for radiologists and non-radiologists with different clinical background and levels of expertise in prostate MRI, ranging from radiologists with more than 30 years of prostate MR reporting to urologists and computer scientists who had seen only a few prostate MR exams before the course.

Our overall goal of providing an intervention to educate a diverse audience has been shown to be successful.

PI-QUAL can be understood and applied by participants and we hope, be an important contribution when used in the clinic to assess the overall value of prostate MRI.

In particular, the participants’ average accuracy in evaluating image quality (suboptimal *vs* optimal) significantly increased after a didactic lecture and a hands-on workshop.

Numerous efforts have been made to improve MR image quality in different organs other than the prostate, including the heart [13], liver [14], brain [15], and breast [16], as radiologists should become familiar with the requirements for good-quality MRI and strive to meet the expected standards to enhance patient quality and safety.

It has already been shown that dedicated teaching courses improve readers’ performances in MRI [17, 18]. The widespread use of this technique in prostate cancer has resulted in high variability of image quality for each sequence and clinical decisions can be compromised if the scans are not acquired at the highest standard [3].

A consensus report by the European Society of Urogenital Radiology (ESUR) and the European Association of Urology - Section of Urologic Imaging (ESUI) has pointed out the vast inconsistency in the conduction of prostate MRI [4].

In this pilot study, the study participants who evaluated the image quality had different backgrounds and different levels of experience in prostate MRI as reported in Table 2. The participants ranged from very experienced radiologists (i.e., reporting more than 1,500 prostate MR scans) scans to other expert clinicians (i.e., urologists) or physicists/computer scientists/engineers with very little experience.

All participants achieved very high and significant accuracy levels in the evaluation of image quality after the course, both overall and when split according to their experience (with the highest values for the experienced participants).

It is important to mention that MR examinations require careful oversight and attention to detail by radiographers and technologists. When starting out in many practices, it can be challenging, as the experience may be lacking. Prostate MR exams from non-academic centres can be variable and can be of suboptimal imaging quality due to a lack of awareness of the quality [9]. In contradistinction, quality is more consistent in high-volume academic centres where there are dedicated teams to oversee quality and radiologists are often involved in clinical research activities relating to prostate cancer imaging. Also, centres where there are large active biopsy programmes will also have more consistent quality as they often provide the MR images for MR-ultrasound fusion biopsy devices [9].

PI-QUAL is the first such effort to alert the MR imaging community of the importance of prostate MR quality. Therefore, the widespread understanding, awareness, application, and validation of this approach is necessary for future adoption [19–21], and the format of this course, with pre- and post-teaching assessment of MR image quality, could be very helpful to disseminate the use of the PI-QUAL score and promote the awareness of the importance of image quality in prostate MRI [22–27].

We believe that the results of our pilot study represent a first step in the right direction, although we should point out that the very high AUC after teaching does not reflect a true experience in evaluating prostate MR images, as this is something that can be achieved only after viewing several prostate MR scans.

This study has limitations; foremost is the small number of participants and MR examinations, but as a pilot study, it provided an impetus for more work with larger numbers.

In addition to this, the reference standard was based on the scores given by a single reader, although highly experienced in the evaluation of image quality by means of PI-QUAL. Given the promising results in terms of inter-reader agreement of PI-QUAL [1, 22, 27], future studies should include the readings from at least two experts as a reference standard.

Another may be the limitation of “one test for all,” as we noted that our participants are diverse, and it may be wise to consider tailoring the PI-QUAL assessment to fit the experience and needs of the different groups. Clearly, radiologists will assess a study with a view to interpretation, whereas urologists will assess quality to determine how valid is the result. We believe that future courses like this should be encouraged.

It should be noted that the PI-QUAL primer [10] was conceived to be used both by clinicians (whose main task is to rule in and rule out the presence of clinically significant prostate cancer, and to target the lesions at biopsy) and by physicists, computer scientists, and radiographers who are involved in the acquisition of adequate prostate MR images at different levels (i.e., from setting up the machine to positioning the patient and injecting intravenous contrast at the right time and speed).

It is crucial to reduce the variability in the conduction and quality of prostate MRI so that clinicians can be confident to use it in the prostate diagnosis and treatment pathways, and although there is plenty of useful teaching material on prostate MRI for self-learning available online, the results from our pilot study reiterate the importance of dedicated hands-on training courses for the evaluation of prostate MR image quality.

In conclusion, we believe that a combination of simultaneous lectures and practical workshops can educate and improve the application of PI-QUAL for prostate MRI.

Also, we hope that our initial results from this teaching fellowship, along with those from the other experience [28], represent fertile ground for the widespread use of such courses for other initiatives and that this paper could act as a source of inspiration for future applicants.

## Supplementary information

ESM 1 (DOCX 34 kb)

## Abbreviations

AUC: Area under the curve
CI: Confidence intervals
COVID-19: Coronavirus disease 19
DICOM: Digital Imaging and Communications in Medicine
ESOR: European School of Radiology
ESUI: European association of urology Section of Urologic Imaging
ESUR: European Society of Urogenital Radiology
mpMRI: Multiparametric magnetic resonance imaging
PI-QUAL: Prostate Imaging QUALity
PI-RADS: Prostate Imaging Reporting and Data System
ROC: Receiver operating characteristic

## Declarations

### Guarantor

The scientific guarantor of this publication is Dr. Francesco Giganti.

### Statistics and biometry

One of the authors (AA) has significant statistical expertise.

### Methodology

• retrospective

• experimental

• multi-centre study
