# Supplementary material for: Promoting the use of the PI-QUAL score for prostate MRI quality: results from the ESOR Nicholas Gourtsoyiannis teaching fellowship
Source: Eur Radiol. 2022 Jun 30;33(1):461–71. doi: 10.1007/s00330-022-08947-5 (PMC9244244; doi:10.1007/s00330-022-08947-5)
Supplement: Supplementary file 1 — (DOCX 34 kb) [file 330_2022_8947_MOESM1_ESM.docx]

**Supplementary Table 1** – Reference standard for the 10 cases included in the pre-teaching cohort according to the PI-QUAL scoring sheet.

| Pre-course assessment | | | | | | | | | | |
| --- | --- | --- | --- | --- | --- | --- | --- | --- | --- | --- |
|  | Scan 1 | Scan 2 | Scan 3 | Scan 4 | Scan 5 | Scan 6 | Scan 7 | Scan 8 | Scan 9 | Scan 10 |
| T2-WI |  |  |  |  |  |  |  |  |  |  |
| Axial plane | ✔ | ✔ | ✔ | ✔ | ✔ | ✔ | ✔ | ✔ | ✔ | ✔ |
| Sagittal or coronal plane | ✔ | ✔ | ✔ | ✔ | ✔ | ✔ | ✔ | ✔ | ✔ | ✔ |
| Adequate field of view | ✔ | ✔ |  | ✔ |  | ✔ | ✔ | ✔ |  | ✔ |
| Adequate in-plane resolution | ✔ | ✔ |  | ✔ |  | ✔ |  | ✔ |  | ✔ |
| Adequate slice thickness | ✔ | ✔ |  | ✔ | ✔ | ✔ | ✔ | ✔ | ✔ | ✔ |
| Z-axis correctly positioned | ✔ | ✔ | ✔ | ✔ |  | ✔ | ✔ | ✔ | ✔ | ✔ |
| Capsule clearly delineated | ✔ | ✔ | ✔ | ✔ | ✔ | ✔ | ✔ | ✔ | ✔ | ✔ |
| SVs clearly delineated | ✔ | ✔ | ✔ | ✔ | ✔ | ✔ | ✔ | ✔ | ✔ | ✔ |
| EDs clearly delineated | ✔ | ✔ |  | ✔ |  | ✔ |  | ✔ | ✔ | ✔ |
| NVBs clearly delineated | ✔ | ✔ | ✔ | ✔ |  | ✔ | ✔ | ✔ | ✔ | ✔ |
| Sphincter muscle clearly delineated | ✔ | ✔ | ✔ | ✔ |  | ✔ |  | ✔ |  | ✔ |
| Absence of artefacts | ✔ | ✔ | ✔ | ✔ |  | ✔ | ✔ | ✔ | ✔ | ✔ |
| Is T2-WI of diagnostic quality? | ✔ | ✔ | ✔ | ✔ |  | ✔ | ✔ | ✔ | ✔ | ✔ |
| DWI |  |  |  |  |  |  |  |  |  |  |
| Axial plane matching T2-WI | ✔ | ✔ | ✔ |  | ✔ | ✔ | ✔ | ✔ | ✔ | ✔ |
| Adequate field of view | ✔ | ✔ |  |  | ✔ | ✔ | ✔ | ✔ | ✔ | ✔ |
| Adequate in-plane resolution | ✔ | ✔ | ✔ |  |  | ✔ |  | ✔ |  | ✔ |
| Adequate slice thickness | ✔ |  | ✔ | ✔ | ✔ | ✔ | ✔ | ✔ |  |  |
| Multiple [> 2] *b* values acquired | ✔ | ✔ | ✔ | ✔ | ✔ | ✔ | ✔ | ✔ | ✔ | ✔ |
| High *b* value (synthesised or acquired) | ✔ | ✔ |  | ✔ |  | ✔ | ✔ | ✔ | ✔ | ✔ |
| Adequate ADC map | ✔ | ✔ | ✔ |  |  | ✔ |  | ✔ | ✔ | ✔ |
| Absence of artefacts | ✔ | ✔ | ✔ |  |  | ✔ |  | ✔ |  | ✔ |
| Is DWI of diagnostic quality? | ✔ | ✔ |  |  |  | ✔ |  |  | ✔ | ✔ |
| DCE |  |  |  |  |  |  |  |  |  |  |
| Axial plane matching T2-WI | ✔ | ✔ | ✔ |  | ✔ | ✔ | ✔ | ✔ | ✔ | ✔ |
| Adequate field of view | ✔ | ✔ |  |  | ✔ |  |  | ✔ | ✔ | ✔ |
| Adequate in-plane resolution | ✔ | ✔ |  |  |  |  |  |  | ✔ |  |
| Adequate slice thickness | ✔ | ✔ |  |  | ✔ |  | ✔ |  | ✔ | ✔ |
| Pre-contrast T1-WI available | ✔ | ✔ | ✔ |  | ✔ |  |  | ✔ | ✔ | ✔ |
| Fat suppression/subtraction | ✔ | ✔ |  |  |  |  | ✔ |  | ✔ | ✔ |
| Adequate temporal resolution | ✔ | ✔ |  |  | ✔ | ✔ |  |  | ✔ | ✔ |
| Adequate total observation rate | ✔ | ✔ | ✔ | ✔ | ✔ | ✔ | ✔ | ✔ | ✔ | ✔ |
| Capsular vessels clearly delineated | ✔ | ✔ |  |  | ✔ |  |  |  | ✔ | ✔ |
| Vessels in the Alcock’s canal clearly delineated | ✔ | ✔ |  |  | ✔ |  |  |  | ✔ | ✔ |
| Absence of artefacts | ✔ | ✔ |  |  | ✔ |  |  |  | ✔ | ✔ |
| Is DCE of diagnostic quality? | ✔ | ✔ |  |  | ✔ |  |  |  | ✔ | ✔ |
| **PI-QUAL score** | **5** | **4** | **2** | **2** | **2** | **4** | **2** | **2** | **3** | **4** |

Legend: T2-WI: T2-weighted imaging; SVs: seminal vesicles; EDs: ejaculatory ducts; NVBs: neurovascular bundles; DWI: diffusion-weighted imaging; ADC: apparent diffusion coefficient; DCE: dynamic contrast enhanced; T1-WI: T1-weighted imaging; PI-QUAL: Prostate Image Quality.

**Supplementary Table 2** – Reference standard for the 10 cases included in the post-teaching cohort according to the PI-QUAL scoring sheet.

| Post-course assessment | | | | | | | | | | |
| --- | --- | --- | --- | --- | --- | --- | --- | --- | --- | --- |
|  | Scan 1 | Scan 2 | Scan 3 | Scan 4 | Scan 5 | Scan 6 | Scan 7 | Scan 8 | Scan 9 | Scan 10 |
| T2-WI |  |  |  |  |  |  |  |  |  |  |
| Axial plane | ✔ | ✔ | ✔ | ✔ | ✔ | ✔ | ✔ | ✔ | ✔ | ✔ |
| Sagittal or coronal plane | ✔ | ✔ | ✔ | ✔ | ✔ | ✔ | ✔ | ✔ | ✔ | ✔ |
| Adequate field of view | ✔ | ✔ | ✔ | ✔ |  | ✔ | ✔ |  |  | ✔ |
| Adequate in-plane resolution | ✔ | ✔ | ✔ |  |  | ✔ | ✔ |  |  | ✔ |
| Adequate slice thickness | ✔ | ✔ |  | ✔ |  | ✔ | ✔ |  | ✔ | ✔ |
| Z-axis correctly positioned | ✔ | ✔ | ✔ | ✔ |  | ✔ | ✔ | ✔ |  | ✔ |
| Capsule clearly delineated | ✔ | ✔ | ✔ | ✔ |  | ✔ | ✔ | ✔ | ✔ | ✔ |
| SVs clearly delineated | ✔ | ✔ | ✔ |  |  | ✔ | ✔ | ✔ |  | ✔ |
| EDs clearly delineated | ✔ | ✔ | ✔ |  |  | ✔ | ✔ |  |  | ✔ |
| NVBs clearly delineated | ✔ | ✔ | ✔ | ✔ |  | ✔ | ✔ | ✔ |  | ✔ |
| Sphincter muscle clearly delineated | ✔ | ✔ | ✔ | ✔ |  | ✔ | ✔ | ✔ |  | ✔ |
| Absence of artefacts | ✔ | ✔ | ✔ |  | ✔ | ✔ | ✔ | ✔ |  | ✔ |
| Is T2-WI of diagnostic quality? | ✔ | ✔ | ✔ | ✔ |  | ✔ | ✔ | ✔ |  | ✔ |
| DWI |  |  |  |  |  |  |  |  |  |  |
| Axial plane matching T2-WI | ✔ | ✔ | ✔ | ✔ | ✔ | ✔ | ✔ | ✔ | ✔ | ✔ |
| Adequate field of view | ✔ | ✔ |  | ✔ |  | ✔ | ✔ |  |  | ✔ |
| Adequate in-plane resolution | ✔ | ✔ | ✔ | ✔ |  | ✔ | ✔ |  | ✔ | ✔ |
| Adequate slice thickness | ✔ | ✔ | ✔ | ✔ |  | ✔ | ✔ |  |  | ✔ |
| Multiple [> 2] *b* values acquired | ✔ | ✔ | ✔ | ✔ | ✔ | ✔ | ✔ | ✔ |  | ✔ |
| High *b* value (synthesised or acquired) |  | ✔ | ✔ | ✔ | ✔ | ✔ | ✔ |  | ✔ | ✔ |
| Adequate ADC map | ✔ | ✔ |  | ✔ |  |  | ✔ |  |  | ✔ |
| Absence of artefacts |  | ✔ |  | ✔ |  |  | ✔ |  |  | ✔ |
| Is DWI of diagnostic quality? | ✔ | ✔ |  | ✔ |  |  | ✔ |  |  | ✔ |
| DCE |  |  |  |  |  |  |  |  |  |  |
| Axial plane matching T2-WI | ✔ | ✔ | ✔ | ✔ | ✔ | ✔ | ✔ | ✔ | ✔ | ✔ |
| Adequate field of view |  | ✔ | ✔ | ✔ | ✔ | ✔ | ✔ |  |  | ✔ |
| Adequate in-plane resolution | ✔ | ✔ | ✔ | ✔ |  | ✔ | ✔ |  |  | ✔ |
| Adequate slice thickness | ✔ |  | ✔ | ✔ |  |  | ✔ |  |  | ✔ |
| Pre-contrast T1-WI available | ✔ | ✔ | ✔ | ✔ |  | ✔ | ✔ | ✔ |  | ✔ |
| Fat suppression/subtraction | ✔ | ✔ | ✔ | ✔ | ✔ | ✔ | ✔ | ✔ | ✔ | ✔ |
| Adequate temporal resolution |  | ✔ | ✔ | ✔ |  | ✔ | ✔ |  |  | ✔ |
| Adequate total observation rate | ✔ | ✔ | ✔ | ✔ | ✔ | ✔ | ✔ | ✔ |  | ✔ |
| Capsular vessels clearly delineated | ✔ | ✔ | ✔ | ✔ |  | ✔ | ✔ | ✔ | ✔ | ✔ |
| Vessels in the Alcock’s canal clearly delineated | ✔ | ✔ | ✔ | ✔ |  | ✔ | ✔ | ✔ | ✔ | ✔ |
| Absence of artefacts | ✔ | ✔ | ✔ | ✔ |  | ✔ | ✔ | ✔ |  | ✔ |
| Is DCE of diagnostic quality? | ✔ | ✔ | ✔ | ✔ |  | ✔ | ✔ |  |  | ✔ |
| **PI-QUAL score** | **4** | **4** | **4** | **4** | **1** | **4** | **5** | **2** | **1** | **5** |

Legend: T2-WI: T2-weighted imaging; SVs: seminal vesicles; EDs: ejaculatory ducts; NVBs: neurovascular bundles; DWI: diffusion-weighted imaging; ADC: apparent diffusion coefficient; DCE: dynamic contrast enhanced; T1-WI: T1-weighted imaging; PI-QUAL: Prostate Image Quality.
